# The Menstrual Cycle Alters Resting-State Cortical Activity: A Magnetoencephalography Study

**DOI:** 10.3389/fnhum.2021.652789

**Published:** 2021-07-26

**Authors:** Rika Haraguchi, Hideyuki Hoshi, Sayuri Ichikawa, Mayuko Hanyu, Kohei Nakamura, Keisuke Fukasawa, Jesús Poza, Víctor Rodríguez-González, Carlos Gómez, Yoshihito Shigihara

**Affiliations:** ^1^Clinical Laboratory, Kumagaya General Hospital, Kumagaya, Japan; ^2^Precision Medicine Centre, Hokuto Hospital, Obihiro, Japan; ^3^Department of Gynaecology, Kumagaya General Hospital, Kumagaya, Japan; ^4^Genomics Unit, Keio Cancer Centre, Keio University School of Medicine, Minato, Japan; ^5^Biomedical Engineering Group, Higher Technical School of Telecommunications Engineering, University of Valladolid, Valladolid, Spain; ^6^Centro de Investigación Biomédica en Red en Bioingeniería, Biomateriales y Nanomedicina (CIBER-BBN), Valladolid, Spain; ^7^Instituto de Investigación en Matemáticas (IMUVA), University of Valladolid, Valladolid, Spain; ^8^Precision Medicine Centre, Kumagaya General Hospital, Kumagaya, Japan

**Keywords:** menstrual cycle, quantitative spectral parameters, regional oscillatory intensity, representative value, spontaneous neural oscillation

## Abstract

Resting-state neural oscillations are used as biomarkers for functional diseases such as dementia, epilepsy, and stroke. However, accurate interpretation of clinical outcomes requires the identification and minimisation of potential confounding factors. While several studies have indicated that the menstrual cycle also alters brain activity, most of these studies were based on visual inspection rather than objective quantitative measures. In the present study, we aimed to clarify the effect of the menstrual cycle on spontaneous neural oscillations based on quantitative magnetoencephalography (MEG) parameters. Resting-state MEG activity was recorded from 25 healthy women with normal menstrual cycles. For each woman, resting-state brain activity was acquired twice using MEG: once during their menstrual period (MP) and once outside of this period (OP). Our results indicated that the median frequency and peak alpha frequency of the power spectrum were low, whereas Shannon spectral entropy was high, during the MP. Theta intensity within the right temporal cortex and right limbic system was significantly lower during the MP than during the OP. High gamma intensity in the left parietal cortex was also significantly lower during the MP than during the OP. Similar differences were also observed in the parietal and occipital regions between the proliferative (the late part of the follicular phase) and secretory phases (luteal phase). Our findings suggest that the menstrual cycle should be considered to ensure accurate interpretation of functional neuroimaging in clinical practice.

## Introduction

Advancements in neuroimaging techniques have led to their widespread adoption in the clinical examination of functional brain diseases (Brammer, 2009; Khanna et al., 2015; Hoshi and Shigihara, 2020; Tanoue et al., 2021). In functional neuroimaging, brain function is primarily assessed based on changes in metabolism (i.e., position emission tomography), blood flow (i.e., functional magnetic resonance imaging, fMRI), or electrophysiology [i.e., magnetoencephalography (MEG) and electroencephalography (EEG)]. Both MEG and EEG record brain activity in terms of “spontaneous neural oscillations,” which are altered by diverse brain disorders such as epilepsy and dementia (Fernández et al., 2013; Ahmed and Rutka, 2016). However, physiological factors such as age and sex can also affect the frequency and regional patterns of spontaneous neural oscillations, representing potential confounding factors in relevant studies (Dustman et al., 1993; Vysata et al., 2012; Barry and De Blasio, 2017; Hoshi and Shigihara, 2020).

**FIGURE 1:**
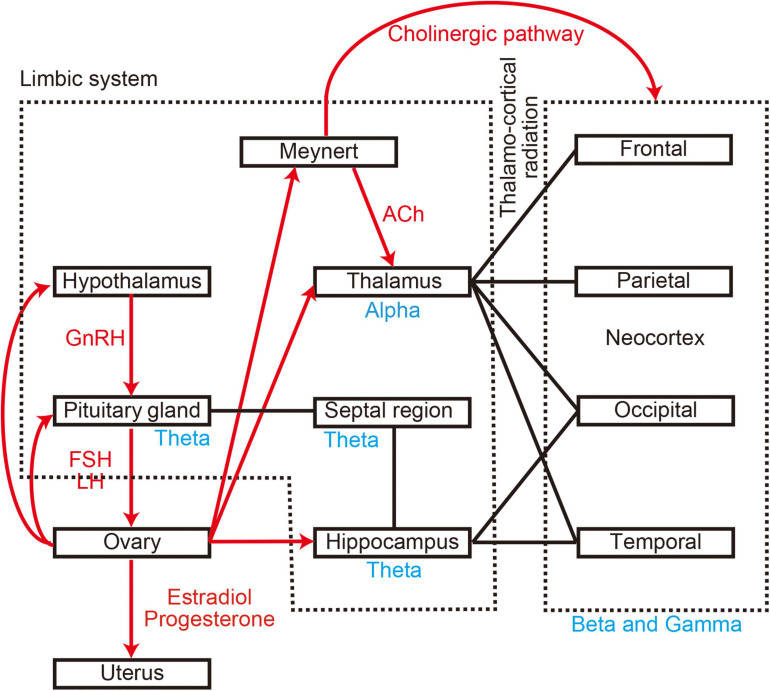
Anatomical and molecular relationship between brain regions. Black lines represent anatomical connections. Red arrows represent molecular connections. Blue words indicate generators of oscillatory activity. GnRH, gonadotropin-releasing hormone; FSH, follicle-stimulating hormone; LH, luteinising hormone; Ach, acetylcholine.

Several studies have indicated that the menstrual cycle also alters brain activity (Lindsley and Rubenstein, 1937; de Barenne and Gibbs, 1942; Creutzfeldt et al., 1976; Becker et al., 1982; Bazanova et al., 2014). The menstrual cycle is produced by a network between the hypothalamus, pituitary gland, and ovaries, which interact via sex hormones such as gonadotropin-releasing hormone (GnRH), follicle-stimulating hormone (FSH), luteinising hormone (LH), oestradiol, and progesterone (Creutzfeldt et al., 1976; Franz, 1988; Hawkins and Matzuk, 2008; Brötzner et al., 2014; see Figure 1). The cycle causes changes at the functional, molecular, and structural levels of the brain and affects both emotion and cognition (Sacher et al., 2013). Functional changes appear as a slowing down or attenuation of spontaneous neural oscillations during the menstrual cycle, and are accompanied by molecular-related changes in sex hormone levels such as oestradiol and progesterone (Creutzfeldt et al., 1976; Becker et al., 1982; Bazanova et al., 2014; Brötzner et al., 2014). These two alterations have recently attracted attention because they are often observed in patients with cognitive dysfunction, including those with dementia (Ferna and Hornero, 2006; Poza et al., 2007; López et al., 2014; Shigihara et al., 2020a). In the case of cognitive dysfunction, damage to the nucleus basalis of Meynert in the basal forebrain leads to decreased cholinergic input to the cortices, thereby leading to changes in spontaneous neural oscillations (Gratwicke et al., 2013; Figure 1). Although the hormones primarily responsible for these changes differ between the menstrual cycle and cognitive dysfunction, evidence suggests a shared neural basis given the interactions between the cholinergic system and oestradiol (Newhouse and Dumas, 2015). Despite the available evidence, most previous studies were limited because they were based on visual inspection (i.e., a subjective analysis). Given that neural signal processing methods have dramatically improved over the last two decades, further studies are required to explore the influence of the menstrual cycle on neural patterns.

During the last few decades, researchers have proposed several parameters for characterising the spectral features of neural oscillations, including median frequency (MF), individual alpha frequency (IAF), and Shannon entropy (SE) (Poza et al., 2007). These parameters can be calculated mechanically from the power spectral density (PSD) of the MEG signals. Thus, they are objective and independent of the skill or experience of the examiner. This is of paramount importance in clinical practice, as it improves the reproducibility, replicability, and reliability of the research findings. These parameters enable intuitive quantification of the diverse properties of brain activity. Furthermore, they are sensitive to changes in cognitive performance and have been highlighted as potential biomarkers for cognitive disorders such as dementia (Poza et al., 2007; Hughes et al., 2019). Indeed, our hospital group utilises these MEG parameters (MF, IAF, and SE) as clinical tools for evaluating hundreds of patients with dementia, stroke, and epilepsy each year. However, no such analyses have revealed how the menstrual cycle affects spontaneous neural oscillations.

Advances in signal processing are not limited to representative parameters of neural oscillations (MF, IAF, and SE). Improvements in processing speed have allowed us to calculate the regional oscillatory intensity of brain activity. Each brain region is associated with specific functions (i.e., “functional specialisation” Mahon and Cantlon, 2011), and there is often a direct link between the damaged cortex and clinical symptoms. Consequently, regional oscillatory intensity gives us information relevant to neurological diseases (Fernández et al., 2013; Sakamoto et al., 2016; Pratt et al., 2017; Shigihara et al., 2020b). Previous studies have revealed that the menstrual cycle affects regional brain activity at both the functional (Bayer et al., 2014; Albert et al., 2015; Pletzer et al., 2019; Weis et al., 2019) and structural levels (Hagemann et al., 2011; Pletzer et al., 2018). fMRI studies have indicated that the menstrual cycle modifies brain activity in various regions, such as the prefrontal cortex, limbic system, hippocampus, amygdala, and striatum (Albert et al., 2015; Pletzer et al., 2019; Weis et al., 2019). Anatomical MRI studies have also reported that grey matter volume changes along the menstrual cycle, reaching its maximum value at the time of ovulation (Hagemann et al., 2011). In contrast, the right parahippocampal/fusiform gyrus reaches its maximum volume during the early follicular phase of the cycle (Pletzer et al., 2018). Although these findings suggest that regional oscillatory intensity should also vary along the menstrual cycle, this hypothesis remains to be verified.

**FIGURE 2:**
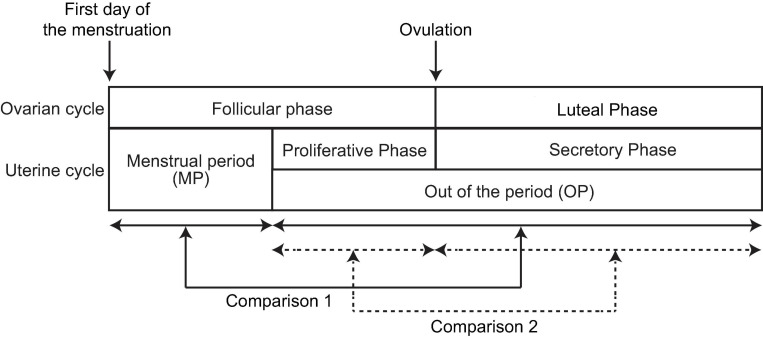
Periods and phases of the menstrual cycle. The first row defines the ovarian cycle, whereas the second row defines the uterine cycle. One cycle includes two periods: the menstrual period (MP) and the period outside of menstruation (OP). The latter is further broken down into two phases: the proliferative and secretory phases. The follicular phase comprises the MP and proliferative phase, whereas the secretory phase is equivalent to the luteal phase. In the present study, neural activity was compared in two ways: (i) between the MP and OP (Comparison 1, solid arrows) and (ii) between the proliferative and secretory phases (Comparison 2, broken arrows).

Therefore, in the present study, we aimed to clarify the effect of the menstrual cycle on spontaneous neural oscillations based on quantitative parameters rather than visual inspection, with the goal of providing personalised clinical assessments (i.e., “precision medicine”). More specifically, we aimed to (i) update previous findings related to the “slowing down and attenuation of oscillatory intensity” using quantitative parameters (Study Goal 1) and (ii) identify the role of the regions responsible for the observed changes in resting-state brain activity (Study Goal 2). To achieve these goals, we measured resting-state spontaneous neural oscillations in 25 healthy women using MEG once during the menstrual period (MP) (i.e., “menses”) and once outside of the menstrual period (OP) (see Figure 2). The representative values of the spontaneous neural oscillations were calculated using established algorithms and compared along the cycle to standardise changes in these values.

## Materials and Methods

### Participants and Ethics

Twenty-five healthy women (mean age ± standard deviation: 28.4 ± 8.0 years; age range: 22–48 years) were enrolled in the present study. The length of their menstrual cycle ranged between 25 and 36 days (29.6 ± 2.7 days), and none of them used oral contraceptives. All participants were staff members at Kumagaya General Hospital, and their health condition was checked annually in accordance with Japanese law (Industrial Safety and Health Act). The present study was conducted in accordance with the ethical principles of the Declaration of Helsinki and was approved by the Ethics Committee of Kumagaya General Hospital (approval number: 14). Written informed consent was obtained from each participant during enrolment.

### Procedure

All participants visited the MEG room twice: once during the MP and once during the OP (see Figure 2). For eight participants, MEG activity was acquired during the MP first. For the remaining 17 participants, MEG activity was acquired during the OP first. The interval between the two MEG scans ranged from 6 to 133 days (mean ± standard deviation: 41.3 ± 43.4 days). For the OP, participants were asked to report the beginning of the last MP and the average length of their cycle to identify the phase of the cycle (proliferative phase or secretory phase) on the day of MEG recording. One participant failed to report the last MP, and her phase was not identified.

The cycle is generally defined in two ways: ovarian cycle and uterine cycle (Figure 2). These two cycles start with the first day (beginning) of the MP, with ovulation occurring in the middle of the cycle. In the ovarian cycle, the period from the first day of menstruation to the day of ovulation is known as the follicular phase, which lasts 16.9 days on average (Bull et al., 2019). The interval from the day of ovulation to the next MP is known as the luteal phase and lasts 12.4 days on average. Thus, we assumed that the menstrual cycle can be divided into follicular and luteal phases using the ratio 16.9:12.4, regardless of the length of the menstrual cycle (Bull et al., 2019). We used this ratio to estimate the phase of the cycle (follicular or luteal) on the recording day. In the uterine cycle, the MP and subsequent proliferative phase (i.e., “pre-ovulatory phase”) occupy the follicular phase of the ovarian cycle. The secretory phase of the uterine cycle corresponds to the luteal phase of the ovarian cycle. Thus, the OP consists of the proliferative phase and secretory phase of the uterine cycle.

In the present study, we performed two different comparisons to assess changes in the spectral content of neural oscillatory MEG activity along the menstrual cycle. First, we compared spontaneous neural oscillations between the MP and OP (Comparison 1 in Figure 2, within-participant design). Second, we compared spontaneous neural oscillations between the proliferative phase (the later part of the follicular phase) and secretory phase (luteal phase) within the OP (Comparison 2 in Figure 2, between-participant design). Eleven participants visited the MEG room during the proliferative phase, while 13 participants visited during the secretory phase. Data from one participant was used for Comparison 1 only and was excluded from Comparison 2 because the participant failed to report the last MP, and the phase could not be identified.

### MEG Scanning Details

Spontaneous neural oscillations were recorded for 5 min using a 160-channel whole-head type MEG system (RICOH160-1; RICOH Co., Ltd., Tokyo, Japan) in a magnetically shielded room. During the scan, participants were asked to remain calm in the supine position with their eyes closed. The scanning conditions were controlled to be as consistent and comfortable for participants as possible. The sensors and reference coils were gradiometers, with diameters of 15.5 and 50 mm at the baseline, respectively. Each pair of sensor coils was separated by a distance of 23 mm. The sampling frequency was 2,000 Hz, and a 500-Hz low-pass filter was used during recording. To co-register MEG source images with structural brain images acquired using canonical MRI, three fiducial magnetic marker coils were placed on each participant’s face (5 mm above the nasion and bilaterally 10 mm in front of the tragus) during the MEG scan.

### MEG Data Analysis

MEG data were pre-processed offline using the software package SPM-12 (Wellcome Trust Centre for Neuroimaging, London, United Kingdom^1^) and the MEAW system^2^. Two types of standard MEG analyses were applied: sensor-level and source-level analyses. Each method is associated with advantages over the other (Shigihara et al., 2020a). Sensor-level analysis produces mathematically reliable results and is less time-consuming than source-level analysis (i.e., taking only a few minutes), making it valuable for clinical practice. Sensor-level analysis is also sensitive to global changes in brain activity, allowing researchers to examine individual differences in spatial distribution. In the present study, sensor-level analysis was used to obtain representative values of the PSD and to replicate the previous finding that spontaneous neural oscillations slow during the MP (Lindsley and Rubenstein, 1937; de Barenne and Gibbs, 1942; Creutzfeldt et al., 1976; Becker et al., 1982; Brötzner et al., 2014) (Study Goal 1). In contrast, source-level analysis provides information regarding regional brain activity, although it is time-consuming (i.e., taking 30–60 min for a single patient). Source-level analyses were adopted to assess differences in regional changes between two conditions (MP vs. OP, or proliferative phase vs. secretory phase) (Study Goal 2).

#### Sensor-Level Data Processing

Sensor-level analyses were performed in accordance with the protocol described in our previous study (Shigihara et al., 2020a). If necessary, prominent artefacts were manually removed via principal component analysis (PCA) using the MEG analysis software developed by the manufacturer because spectral parameters are sensitive to artefacts. The software is authorised for clinical use by the Ministry of Health, Labour, and Welfare of Japan (equivalent to FDA approval). Most artefacts are caused by silver tooth fillings and eye movements; hence, their frequency is usually low (delta to theta range), which can influence the computation of spectral parameters. In addition, artefacts from outside the brain present as characteristic patterns on contour maps and can lead to unnatural time courses for the PCA components (Gross et al., 2013). Experienced clinicians and technicians can distinguish these artefacts from brain signals based on visual inspection. A 50-Hz band-stop filter was also applied to remove power line noise. Thereafter, three spectral parameters were calculated to summarise different properties of spontaneous neural oscillations: MF, IAF, and SE (Poza et al., 2007). The spectral parameters were computed from the PSD, which was estimated using the Blackman–Tukey method considering non-overlapping 10-s epochs. Afterward, the PSD was normalised between 1 and 70 Hz (PSDn) (Gómez et al., 2013). The first parameter, MF, refers to the median of the distribution represented by the PSDn (i.e., the frequency that splits the PSDn into two halves of equal power) (Poza et al., 2007). The second parameter, IAF, refers to the frequency corresponding to the peak of the PSDn in the extended alpha band (4–15 Hz) (i.e., the dominant alpha activity), which usually appears in human adults in the eyes-closed resting condition (Poza et al., 2007). IAF is useful for describing the loss of neural oscillations in the alpha band (i.e., the “*shift-to-the-left*” of the alpha peak). Finally, SE is an irregularity measure closely related to the concept of order in information theory that quantifies the distribution of the oscillatory components of the PSDn (Poza et al., 2007). These three parameters were calculated for each epoch and MEG sensor, following which they were averaged across epochs and sensor position (left and right). Of note, the side of the sensors was not completely matched with each brain hemisphere because our MEG system was equipped with axial gradiometers. As such, left sensors received some signals from the right hemisphere. However, it should be noted that a previous study demonstrated that there is little signal contamination across regions, and that sensor-level information (i.e., that from left and right sensors) is nearly consistent with source-level information (i.e., that from the left and right hemispheres) (Rodríguez-González et al., 2020). Source-level analysis was performed to accurately determine the source of the signals (see section “Source-Level Analysis”). Finally, the averaged parameters were statistically analysed as described in section “Statistical Analysis”.

#### Source-Level Analysis

The source-level analysis for individual participants (first-level analysis) followed the pipeline used in a previous study (Shigihara et al., 2020b). Continuous MEG signals were divided into non-overlapping 10-s epochs. Because the experimental environment generated a utility frequency, a 50-Hz band-stop filter was applied to the epoched data. These filtered data were then directly used for source-level analyses. To identify the brain regions producing the resting-state-induced components, a source inversion procedure was applied to the delta (0–3 Hz), theta (4–7 Hz), alpha (8–12 Hz), beta (13–25 Hz), and gamma (low-gamma, 26–40 Hz; high gamma, 41–80 Hz) oscillatory components separately, using a maximal smoothness algorithm with a spatially coherent sources model (i.e., the COH algorithm implemented in SPM-12) (Friston et al., 2008). This source localisation algorithm is comparable to those used in standardised low-resolution brain electromagnetic tomography (Pascual-Marqui, 2002). The COH algorithm is a popular source inversion algorithm that is often used in clinical environments (Terakawa et al., 2008; Ray and Bowyer, 2010; Shigihara et al., 2020b). Forward modelling was performed for the whole brain using a single-shell model with canonical MRIs provided by SPM-12. The source inversion and estimation steps were performed by applying filters corresponding to each frequency band (from delta to high gamma). No source priors were used for source estimation. The estimated oscillatory intensity at each frequency band and in each brain region (i.e., regional oscillatory intensity) was saved as a source image file in the NIfTI format and used for the second (group)-level analysis.

To increase the sensitivity to changes in regional neural oscillatory intensity between the two conditions in two ways (Comparisons 1 and 2 in Figure 2), we analysed the data in different regions of interest (ROIs). Five sets of ROI mask images (NIfTI format) were created using the WFU pick atlas^3^ for each hemisphere (left and right) in order to cover the whole cortex: frontal, temporal, parietal, occipital, and limbic system. Oscillatory intensities were averaged within each mask using the SPM function “spm_summarise” for each condition in each participant. Averaged oscillatory intensities in the ROIs (ROI values) were analysed as described in section “Statistical Analysis”.

### Statistical Analysis

We first performed Comparison 1 within each participant. As condition order was not counterbalanced (eight participants were scanned during the MP first while 17 were scanned in reverse order), we examined the effect of order. The three sensor-level spectral parameters were compared between first and second MEG scans using a bootstrapping method, irrespective of the MP or phases. For each parameter, the average difference between the first and second scans was computed via resampling with replacement data across all participants 20,000 times, and the percentage of the resampled average difference larger or smaller than 0 (the smaller value) was taken as the significance level (*p*-value). As we observed statistically significant effects of order, the effects were taken into account in the following statistical analysis using a weighted bootstrapping approach. For this task, the weights (i.e., proportion for resampling) were controlled so that half of the resampled data were derived from the first MEG scan, while the other half were derived from the second scan. This approach resulted in pseudo-counterbalancing after resampling, which allowed us to examine the effects of interest (e.g., difference between phases) while controlling for the effects of order.

Second, sensor-level spectral parameters were compared between the MP and OP (Comparison 1), and between the proliferative and secretory phases (Comparison 2) using a weighted bootstrapping method. For Comparison 1, the average difference between MP and OP was computed via (weighted) resampling with replacement data across all participants 20,000 times, and the percentage of the resampled average difference larger or smaller than 0 (the smaller value) was taken as the significance level (*p*-value). For Comparison 2, the group (i.e., proliferative and secretory phases) average was computed via (weighted) resampling with replacement data across all participants 20,000 times, and the group difference was stored for each iteration. The percentage of the group differences larger or smaller than 0 (the smaller value) was taken as the significance level (*p*-value).

We also examined the relationships between participant age, length of the menstruation cycle, and sensor-level spectral parameters (during the MP, OP, and the difference between the two) using a weighted bootstrapping approach. For each pair of variables, Pearson’s coefficients were calculated via (weighted) resampling with replacement data across all participants 20,000 times. The percentage of the resampled coefficients larger or smaller than 0 (the smaller value) was taken as the significance level (*p*-value).

Finally, we performed Comparisons 1 and 2 for source-level ROI values using the same method used for sensor-level spectral data. The comparisons were made for each ROI and frequency band.

For all statistical analyses, we report the grand mean of the statistical values (e.g., mean value of each condition, standard error of resampled cases, group-difference: *d*, and average correlation coefficient: *r*) across bootstrap iterations, as well as *p*-values. Throughout all statistical examinations, statistically significant *p*-values were determined after controlling the false detection rate (FDR) using the Benjamini and Hochberg method (Benjamini and Hochberg, 1995).

## Results

### Sensor-Level Analysis (Spectral Parameters)

#### Effect of Order

We observed significant effects of order on MF for sensors on the right side (rtMF) and on IAF for sensors on both sides (rtMF: *d* = −0.283, *p* = 0.025; IAF in the left, ltIAF: *d* = −0.199, *p* = 0.027; IAF in the right, rtIAF: *d* = −0.214, *p* = 0.020), indicating that values measured first were higher than those measured second.

#### Comparison 1: MP vs. OP

**FIGURE 3:**
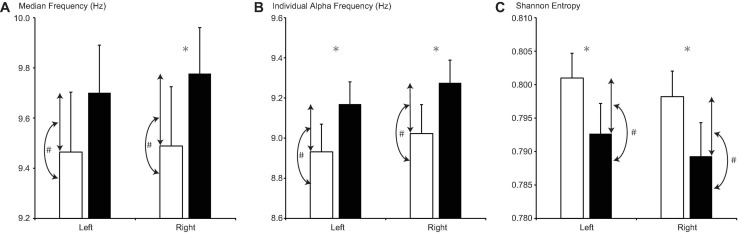
Changes in **(A)** median frequency, **(B)** individual alpha frequency, and **(C)** Shannon entropy between the menstrual period (MP, open columns) and the period outside of menstruation (OP, filled columns). Error bars represent the grand mean of standard errors over bootstrapped iterations. Asterisks (^∗^) indicate statistically significant differences (after false-discovery rate (FDR) correction) between the two periods. Hash tags (#) indicate statistically significant correlations (after FDR correction) between the value of the parameter in each period and its change.

For sensors on both sides, rtMF and IAF were lower during MP than during OP (rtMF: *d* = −0.288, *p* = 0.011; ltIAF: *d* = −0.238, *p* = 0.004; rtIAF: *d* = −0.253, *p* = 0.002; Figure 3). Although MF values for the left side (ltMF) were lower during MP than during OP, the difference did not reach significance after FDR correction (*d* = −0.236, *p* = 0.040). SE values on both sides were higher during MP than during OP (right side, rtSE: *d* = 0.008, *p* = 0.001; left side, ltSE: *d* = 0.009, *p* = 0.002).

For sensors on both sides, the difference between MF values and the difference between IAF values were positively correlated with MF and IAF values during the MP, respectively (ltMF: *r* = 0.593, *p* = 0.006; rtMF: *r* = 0.623, *p* = 0.002; ltIAF: *r* = 0.587, *p* = 0.001; rtIAF: *r* = 0.614, *p* < 0.001), but not during OP. Differences in SE on both sides were negatively correlated with SE during OP (ltSE: *r* = −0.572, *p* < 0.001; rtSE: *r* = −0.635, *p* < 0.001), but not during MP.

#### Comparison 2: Proliferative Phase (Later Part of the Follicular Phase) vs. Secretory Phase (Luteal Phase)

**FIGURE 4:**
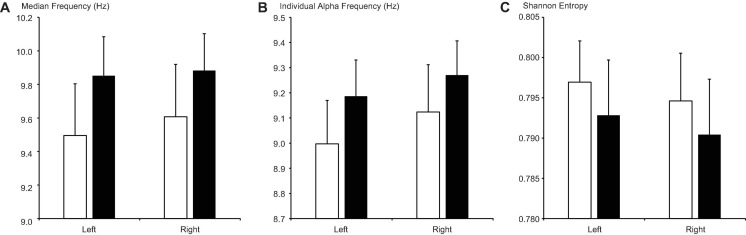
Changes in **(A)** median frequency, **(B)** individual alpha frequency, and **(C)** Shannon entropy between the proliferative phase (open columns) and secretory phase (filled columns). Error bars represent the grand mean of standard errors over bootstrapped iterations.

Although MF and IAF values were higher, while SE values were lower, on both sides during the secretory phase than during the proliferative phase (Figure 4), these differences did not reach significance after FDR correction (ltMF, *p* = 0.190; rtMF, *p* = 0.240; ltIAF, *p* = 0.202; rtIAF, *p* = 0.270; ltSE, *p* = 0.314; rtSE = 0.329).

#### Age and Cycle Length

For sensors on both sides, age was positively correlated with the MF value during the MP (ltMF: *r* = 0.416, *p* = 0.012; rtMF: *r* = 0.398, *p* = 0.018) (Table 1). In addition, age was positively correlated with the change in IAF between MP and OP on the left side (*r* = 0.206, *p* = 0.040). The length of the menstrual cycle was negatively correlated with the IAF value on both sides during the OP (ltIAF: *r* = −0.379, *p* = 0.006; rtIAF: *r* = −0.407, *p* = 0.002). The correlation between participant age and cycle length was not statistically significant (*p* = 0.214). No statistically significant correlations were observed for the other pairs between age/cycle length and spectral parameters (Table 1).

**TABLE 1:** Correlation between spectral parameters and participant age/length of the menstrual cycle (Comparison 1 in sensor-level analysis).

		MF				IAF				SE			
		Left		Right		Left		Right		Left		Right	
		*r*	*p*	*r*	*p*	*r*	*p*	*r*	*p*	*r*	*p*	*r*	*p*
Menstrual period (MP)	Age	0.416	0.012*	0.398	0.018*	0.222	0.086	0.248	0.071	0.104	0.322	0.117	0.281
	Cycle	−0.171	0.150	−0.124	0.247	−0.219	0.083	−0.169	0.155	−0.273	0.100	−0.232	0.107
Outside of the menstrual period (OP)	Age	0.381	0.050	0.310	0.070	0.103	0.207	0.132	0.167	0.008	0.498	−0.032	0.432
	Cycle	−0.120	0.250	−0.213	0.115	−0.379	0.006*	−0.407	0.002*	−0.120	0.260	−0.157	0.185
Change	Age	0.163	0.236	0.275	0.059	0.206	0.040*	0.214	0.049	0.121	0.257	0.180	0.170
	Cycle	−0.116	0.219	0.082	0.296	0.117	0.187	0.216	0.087	−0.134	0.161	−0.015	0.446

r, average correlation coefficient across bootstrap iterations; p, p-values of weighted bootstrapping statistics, * indicates statistically significant correlation after FDR correction.

### Source-Level Analysis (Regional Oscillatory Intensity)

#### Comparison 1: MP vs. OP

**FIGURE 5:**
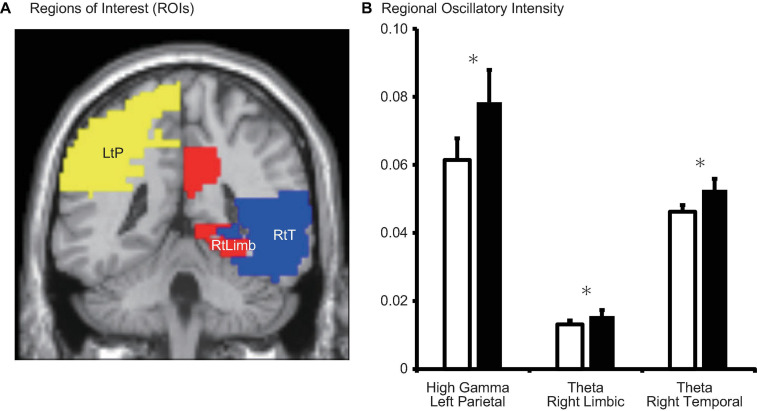
**(A)** Schematic representation of regions of interest (ROIs) and **(B)** regional oscillatory intensity changes during the menstrual period (MP, open columns) and the period outside of menstruation (OP, filled columns). Three parameters with significant changes (after false-discovery rate (FDR) correction) were displayed. Error bars represent the grand mean of standard errors over bootstrapped iterations. LtP, left parietal; RtLimb, right limbic system; RtT, right temporal. Asterisks (*) indicate statistically significant difference after FDR correction.

Theta intensity within the right temporal cortex and right limbic system was significantly lower during the MP than during the OP (right temporal: *d* = −0.007, *p* = 0.010; right limbic system: *d* = −0.002, *p* = 0.003) (Table 2, Figure 5, and Supplementary Figure 1). High gamma intensity in the left parietal cortex was also significantly lower during the MP than during the OP (*d* = −0.017, *p* = 0.037). No other differences in regional oscillatory intensity were observed between the two conditions.

**TABLE 2:** Average change in oscillatory intensity within ROIs and their significance between the menstrual period (MP) and the period outside of menstruation (OP) (Comparison 1 in source-level analysis).

	Frontal				Temporal				Parietal				Occipital				Limbic system			
	Left		Right		Left		Right		Left		Right		Left		Right		Left		Right	
Frequency band	*d*	*p*	*d*	*p*	*d*	*p*	*d*	*p*	*d*	*p*	*d*	*p*	*d*	*p*	*d*	*p*	*d*	*p*	*d*	*p*
Delta	0.000	0.474	0.000	0.350	0.000	0.453	0.000	0.407	−0.002	0.155	−0.001	0.369	−0.002	0.266	−0.001	0.210	−0.001	0.293	−0.001	0.237
Theta	−0.001	0.265	−0.002	0.177	0.002	0.330	−0.007	0.010*	0.006	0.238	0.003	0.254	0.009	0.097	−0.001	0.459	0.001	0.296	−0.002	0.003*
Alpha	0.001	0.171	0.001	0.231	0.000	0.498	0.000	0.491	0.002	0.353	0.005	0.162	−0.002	0.343	0.000	0.482	0.000	0.434	−0.001	0.131
Beta	0.001	0.323	0.000	0.465	0.004	0.129	0.001	0.429	0.007	0.144	0.008	0.086	0.011	0.075	0.006	0.098	0.001	0.270	−0.001	0.249
Low gamma	0.000	0.484	0.001	0.353	0.000	0.474	−0.001	0.428	−0.002	0.391	0.008	0.115	0.006	0.238	0.000	0.461	−0.002	0.079	−0.001	0.213
High gamma	−0.002	0.234	0.004	0.110	−0.004	0.202	0.004	0.180	−0.017	0.037*	0.003	0.327	−0.005	0.204	−0.002	0.355	−0.004	0.136	0.001	0.378

d, average difference across bootstrap iterations; p, p-values of weighted bootstrapping statistics, * indicates statistically significant difference after FDR correction. ROI, region of interest.

#### Comparison 2: Proliferative Phase vs. Secretory Phase

**FIGURE 6:**
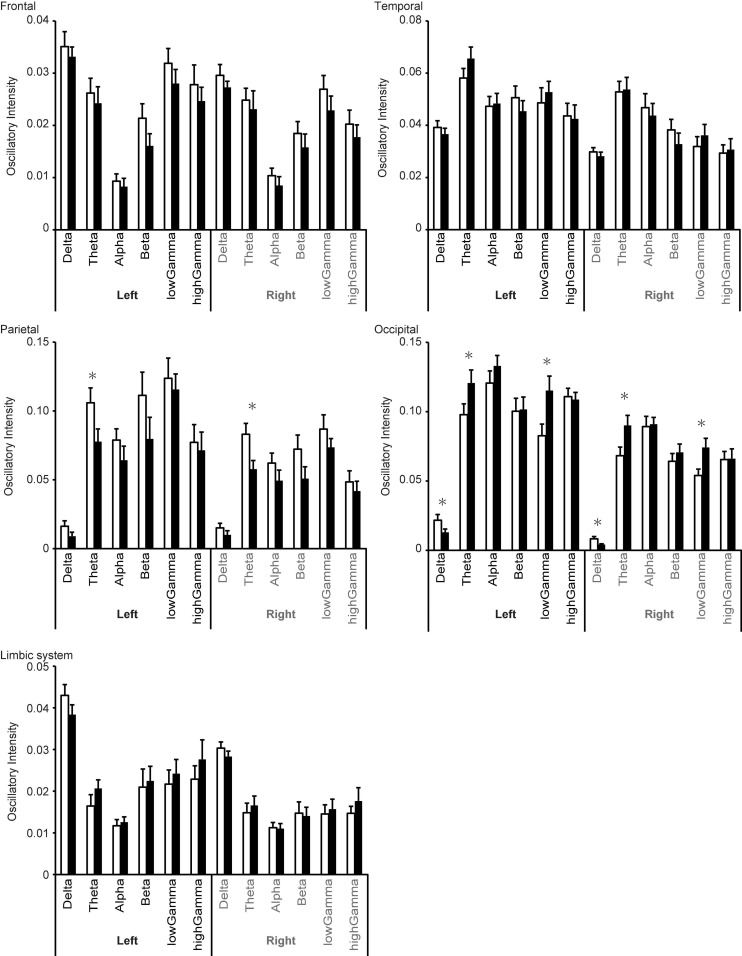
Changes in regional oscillatory intensity during the proliferative (open columns) and secretory (filled columns) phases. Error bars represent the grand mean of standard errors over bootstrapped iterations, whereas asterisks (*) indicate statistically significant differences (after false-discovery rate (FDR) correction).

Significant changes in regional oscillatory intensity were observed on both sides for the parietal and occipital regions. Theta oscillatory intensity was higher on both sides of the parietal region during the proliferative phase than during the secretory phase (Table 3 and Figure 6), while that in the occipital region was lower during the proliferative phase than during the secretory phase. In addition, delta oscillatory intensity was higher while low gamma oscillatory intensity was lower during the proliferative phase than during the secretory phase on both hemispheres. No other differences were observed for any region or frequency band.

**TABLE 3:** Average oscillatory intensity within ROIs and the corresponding statistical differences between the proliferative and secretory phases (Comparison 2 in source-level analysis).

		Left			Right		
	Freq	Proliferative *M*	Secretory *M*	*p*	Proliferative *M*	Secretory *M*	*p*
Frontal	Delta	0.035	0.033	0.299	0.030	0.027	0.168
	Theta	0.026	0.024	0.325	0.025	0.023	0.334
	Alpha	0.009	0.008	0.326	0.010	0.009	0.212
	Beta	0.021	0.016	0.079	0.018	0.016	0.221
	Low gamma	0.032	0.028	0.172	0.027	0.023	0.151
	High gamma	0.028	0.025	0.252	0.020	0.018	0.242
Temporal	Delta	0.039	0.037	0.233	0.030	0.028	0.235
	Theta	0.058	0.066	0.096	0.053	0.054	0.450
	Alpha	0.047	0.048	0.419	0.047	0.044	0.330
	Beta	0.051	0.045	0.203	0.038	0.033	0.185
	Low gamma	0.049	0.053	0.288	0.032	0.036	0.235
	High gamma	0.044	0.042	0.441	0.029	0.031	0.410
Parietal	Delta	0.016	0.009	0.076	0.015	0.010	0.139
	Theta	0.106	0.078	0.033*	0.083	0.058	0.013*
	Alpha	0.079	0.064	0.141	0.062	0.049	0.125
	Beta	0.111	0.080	0.092	0.072	0.051	0.062
	Low gamma	0.124	0.116	0.331	0.087	0.074	0.145
	High gamma	0.077	0.071	0.370	0.048	0.042	0.272
Occipital	Delta	0.022	0.013	0.036*	0.008	0.004	0.006*
	Theta	0.098	0.121	0.038*	0.068	0.090	0.015*
	Alpha	0.121	0.133	0.144	0.089	0.091	0.435
	Beta	0.100	0.102	0.446	0.064	0.071	0.219
	Low gamma	0.083	0.115	0.007*	0.054	0.074	0.008*
	High gamma	0.111	0.109	0.402	0.065	0.066	0.458
Limb	Delta	0.043	0.038	0.099	0.030	0.028	0.151
	Theta	0.016	0.021	0.124	0.015	0.017	0.306
	Alpha	0.012	0.013	0.329	0.011	0.011	0.442
	Beta	0.021	0.022	0.390	0.015	0.014	0.427
	Low gamma	0.022	0.024	0.293	0.015	0.016	0.370
	High gamma	0.023	0.028	0.205	0.015	0.018	0.231

M: average oscillatory intensity within ROI at given frequency in given phase; p, p-values of weighted bootstrapping statistics, asterisks (*) indicate statistically significant difference after FDR correction. ROI, region of interest.

## Discussion

The present study investigated the effect of the menstrual cycle on spontaneous neural oscillations based on quantitative MEG parameters. Our analysis revealed four major findings: (i) MF and IAF were lower during MP than during OP, mainly on the right side of the MEG dewar; (ii) SE was higher during MP than during OP; (iii) theta oscillatory intensity was lower in the right temporal cortex and limbic system during MP than during OP; and (iv) theta oscillatory intensity in the parietal and occipital regions differed between the proliferative and secretory phases.

The menstrual cycle is a fundamental body rhythm that is regulated by five basic hormones: GnRH, FSH, LH, oestradiol, and progesterone (Creutzfeldt et al., 1976; Franz, 1988; Hawkins and Matzuk, 2008; Brötzner et al., 2014; Figure 1). These hormones interact with each other and affect various brain functions in the sensory, cognitive, and emotional domains (Farage et al., 2008; Poromaa and Gingnell, 2014). As neural oscillations are sensitive to changes in brain function (BaŞar, 2013; Mazaheri et al., 2018; Ohki and Takei, 2018), previous studies have already reported that the EEG power spectrum changes across the menstrual cycle (Lindsley and Rubenstein, 1937; de Barenne and Gibbs, 1942; Creutzfeldt et al., 1976; Becker et al., 1982; Bazanova et al., 2014). Most of these previous studies focussed on changes in alpha oscillations, which can easily be identified and evaluated via visual inspection without sophisticated techniques such as signal processing methods (Lewine and Orrison, 1995).

Mean alpha frequency is higher during the luteal phase (secretory phase) than during menstruation, and the change is coupled with an increase in progesterone levels (Creutzfeldt et al., 1976; Becker et al., 1982; Bazanova et al., 2014; Brötzner et al., 2014). Other studies have reported that increased alpha frequency during the luteal phase is associated with changes in cognitive performance (Grandy et al., 2013; Bazanova et al., 2014). Although it is difficult to analyse other neural oscillations (i.e., delta, theta, beta, and gamma) without sophisticated techniques, some studies have also reported that alterations in these rhythms occur during the menstrual cycle. Nonetheless, changes in theta oscillations remain controversial. On one hand, Creutzfeldt et al. (1976) reported that mean theta power was lower during the luteal phase than during the follicular phase. On the other hand, Becker et al. (1982) reported that mean theta power was lower during the periovulatory period and higher during the perimenstrual period. This discrepancy may be explained by methodological and statistical limitations in the earlier study, which was conducted approximately 40–50 years ago. Further evidence indicated that beta intensity was significantly larger during the follicular phase than during the luteal phase (Becker et al., 1982).

Despite these interesting findings, most previous studies regarding the relationship between the menstrual cycle and oscillatory patterns were based on visual inspection rather than signal processing methods. In the present study, we updated these findings by computing three different spectral parameters (MF, IAF, and SE) (Study Goal 1) and regional oscillatory intensities (Study Goal 2). We primally focused on differences between MP and OP (Comparison 1) because they are practically/clinically useful. Clinical practice is based on a good relationship between the patient and clinician. Details regarding menstruation can be difficult to discuss during a patient’s first visit, especially when the clinician is male and when the patient’s chief complaint does not seem to be related. Given its relevance to many conditions, the classification of the MP and OP is essential in clinical practice. In addition, we performed Comparison 2 (proliferative vs. secretory phase) within the OP because the endocrine environment differs between these phases. Although information from Comparison 2 is applicable in limited gynaecological situations, such information can help us to evaluate MEG data more appropriately. Another potential area of interest may be the relationship between hormone levels and dementia, as mentioned in the Introduction. Some research indicates that hormone replacement therapy can attenuate the progression of dementia in women, for example (Moradi et al., 2018; Zhou et al., 2020). MEG may therefore aid in determining the efficacy of such therapy.

### Spectral Parameters (Sensor-Level Analysis)

In the sensor-level analysis, three spectral parameters (MF, IAF, and SE) were used to quantify global changes in neural oscillations related to the menstrual cycle. In this analysis, we aimed to replicate and expand upon the previous visually based finding that alpha frequency is low during the MP and high during the OP, especially during the secretory phase (i.e., luteal phase) (Study Goal 1). The IAF reflects the peak frequency of alpha oscillations. IAF values were lower during the MP than during the OP (Comparison 1). Although they were also lower during the proliferative phase than during the secretory phase, this difference was not significant (Comparison 2). These findings are largely consistent with those of previous studies (Lindsley and Rubenstein, 1937; de Barenne and Gibbs, 1942; Creutzfeldt et al., 1976; Becker et al., 1982).

The MF reflects the oscillatory power balance between high and low frequencies. Although both IAF and MF are affected by changes in peak alpha frequency, MF is also affected by changes in the theta, beta, and gamma bands. As observed for IAF, MF was lower during the MP than during the OP (Comparison 1). Similarly, although MF values were lower during the proliferative phase (the later part of the follicular phase) than during the secretory phase (luteal phase), this difference was not significant (Comparison 2). There are three possible interpretations regarding the change in MF: (i) changes in alpha (peak) frequency, (ii) changes in power balance between high and low frequencies, or (iii) both. These changes are noteworthy because low MF and IAF values are often observed in patients with cognitive dysfunction and dementia (Poza et al., 2007). Previous studies have indicated that some cognitive functions are impaired during the MP, including mental rotation, visuospatial ability, verbal memory, and verbal fluency, although these findings remain controversial (Sommer, 1992; Schöning et al., 2007; Hatta and Nagaya, 2009; Poromaa and Gingnell, 2014; Farrar et al., 2015; Sundström-Poromaa, 2018). Low values of MF and IAF during the MP may reflect impairments in cognitive function due to the menstrual cycle.

The SE is another spectral parameter that represents the irregularity of the distribution of the neural oscillatory components in the PSDn. Our findings indicated that SE was higher during the MP than during the OP (Comparison 1). Although SE was also higher during the proliferative phase than during the secretory phase, this difference was not significant (Comparison 2). Patients with dementia tend to exhibit lower SE values, in addition to low MF and IAF values (Poza et al., 2007). However, our participants exhibited lower MF/IAF values and higher SE values during the MP than during the OP, suggesting that the distribution of neural oscillatory components during MP differs from that related to dementia.

Changes in MF and IAF values between the MP and OP (Comparison 1, marked with # in Figure 3) were positively correlated with their values during the MP, but not during the OP. This result suggests that participants with low values of MF or IAF during the MP exhibited larger changes in these parameters between the MP and OP. Furthermore, despite being a relatively short period within the menstrual cycle, the MP appears to regulate changes in MF or IAF. In addition, the changes in SE were negatively correlated with the value of SE during the OP, but not during the MP, suggesting that participants with low SE values during OP exhibited larger changes in SE between the MP and OP. These results support the notion that MF/IAF and SE reflect different properties of the PSDn.

In accordance with previous findings, MF and IAF exhibited a partial positive correlation with age (Gómez et al., 2013; Hoshi and Shigihara, 2020). However, we observed no significant correlation between participant age and cycle length. We speculate that this is due to bias in participant age: Most participants were in their 20 s, only one was in her 30 s, and four were in their 40 s. Research has indicated that the length of the mensural cycle progressively decreases with age, and that IAF progressively increases with age (Gómez et al., 2013; Bull et al., 2019; Hoshi and Shigihara, 2020), consistent with our finding that the length of the menstrual cycle was negatively correlated with IAF.

Overall, the sensor level analysis successfully replicated the previous findings and provided two additional results: (i) It is plausible that the menstrual cycle affects frequencies other than alpha rhythms; (ii) oscillatory brain patterns during the MP (i.e., increased SE) differ from those associated with dementia.

### Regional Oscillatory Intensity (Source-Level Analysis)

Whereas the sensor-level analysis investigated global changes in neural oscillations in terms of quantitative spectral parameters (MF, IAF, and SE), the source-level analysis provided deeper insight into the brain regions responsible for these changes (Study Goal 2). Interestingly, we observed significant changes in regional oscillatory intensity in the theta and gamma bands rather than the alpha band (Comparison 1 in Figure 5 and Table 2, Comparison 2 in Figure 6 and Table 3). Theta oscillations are generated by a neural network between the hypothalamus, septal region, and hippocampus (Colom, 2006; Figure 1). The hippocampus is a major structure of the limbic system. Given that the hippocampus is rich in oestrogen receptors, it is not surprising that it exhibits changes in function during the menstrual cycle (Woolley et al., 1990; Shughrue and Merchenthaler, 2000; Lisofsky et al., 2015). The hippocampus plays a key role in short-term memory: While the right hippocampus is important for spatial navigation, the left hippocampus is important for verbal memory (Hamid, 2014; Ezzati et al., 2016; Kang et al., 2016). Performance on mental rotation tasks, which are used to investigate spatial navigation, fluctuates with oestradiol and progesterone levels. Previous studies have indicated that task performance is high during the MP and low during the midluteal phase (Hampson et al., 2014; Barel et al., 2019). The hippocampus is located in the medial part of the temporal lobe and is connected with the other parts of the temporal lobe (Rolls, 1989; Wilson et al., 1990; Maller et al., 2019). Consequently, it is reasonable to suggest that theta intensity in the limbic system and temporal region changes between the MP and OP (Comparison 1 in Figures 2, 5). Comparison 1 also revealed that high gamma intensity in the left parietal region differed between the MP and OP. The left parietal lobe is a critical region for verbal processing (Coslett and Schwartz, 2018), which is also modified during the menstrual cycle (Šimič and Santini, 2012; Barel et al., 2019): Women exhibit their best performance on verbal fluency tasks during the menstrual and midluteal phases and worst performance during the early follicular phase (Šimič and Santini, 2012; Barel et al., 2019). Furthermore, gamma oscillatory activity is affected by grey matter volume (Schwarzkopf et al., 2012), and research has indicated that the volume of the left partial lobe changes during the menstrual cycle (Protopopescu et al., 2008). It is plausible that changes in grey matter volume during the menstrual cycle are linked with changes in verbal fluency task performance and gamma oscillatory intensity between the MP and OP (Comparison 1).

Comparison 2 (proliferative phase vs. secretory phase) revealed that the menstrual cycle is associated with changes in oscillatory intensity in the parietal and occipital regions. Changes in theta oscillatory intensity were observed in both regions. These two regions are important for visual perception, which is also affected by the menstrual cycle (Ward et al., 1978). The amplitude of the typical visual-evoked response, which is modulated by the top-down attention system, is larger during the mid-luteal phase (Lusk et al., 2015) than during other phases. Notably, changes in these regions were observed in the theta band but not in the gamma band, which is typically associated with the bottom-up components of visual perception (Tallon-Baudry, 2009; Bastos et al., 2012; Sauseng et al., 2015; Bartoli et al., 2020). Theta oscillations are mainly generated in the hippocampus (Colom, 2006). However, it is unlikely that hippocampal activity directly modifies theta oscillations in these regions. The strongest target of hippocampal connectivity is the temporal lobe, followed by the limbic system and subcortical structures such as the thalamus (Maller et al., 2019). The occipital lobe is the third target, while the parietal lobe exhibits weak direct connectivity with the hippocampus. Thus, it is not plausible that the hippocampus modifies theta oscillations in the parietal and occipital regions without eliciting significant changes in the temporal region.

The hippocampus is connected with the thalamus, which contains receptors for oestradiol, and several studies have demonstrated that the menstrual cycle influences thalamic activity (Sacher et al., 2013; Brinton et al., 2015; Hidalgo-Lopez et al., 2020). In addition, the thalamus is connected with almost all cortices via the thalamocortical radiations (Cunningham et al., 2017; Caspers and Zilles, 2018; George and Das, 2019). These radiations are categorised into four distinct parts based on anatomical position: anterior thalamic radiations, posterior thalamic radiations, superior thalamic radiations, and inferior thalamic radiations (George and Das, 2019). Hence, we assume that parietal and occipital theta oscillatory activity is modulated by the hippocampus via the posterior thalamic radiations, whose target regions are the parietal and occipital regions. This assumption may explain the mechanism underlying the effect of the menstrual cycle on visual perception. The posterior thalamic radiation conveys top-down signals to these two regions from other neural networks such as the attention system using theta oscillations, which serve to provide top-down control (Bastos et al., 2012; Sauseng et al., 2015). Attention exhibits a close relationship with visual perception (Luck and Ford, 1998), and previous studies have indicated that the attention system is affected by the menstrual cycle via progesterone levels (Pletzer et al., 2017). Other studies have indicated that attention is associated with theta oscillations (Hermens et al., 2005; Keller et al., 2017). We speculate that the attention system or relevant networks modulate visual perception via theta oscillations.

In the occipital region, delta oscillatory intensity was higher during the proliferative phase than during the secretory phase. Enhanced delta oscillations in the caudal part of the brain are a typical feature of cognitive dysfunction and dementia (Fernández et al., 2013; Hughes et al., 2019). Although research has indicated that the menstrual cycle alters cognition (Sacher et al., 2013), these alterations are not the same as those observed in patients with dementia. Delta oscillations are associated with network activity rather than local activity (Bastos et al., 2012; Sauseng et al., 2015), and enhanced delta oscillation is associated with loss of cholinergic input from the nucleus basalis of Meynert (Lopes da Silva, 1991; Gratwicke et al., 2013; Figure 1), which contains oestrogen receptors (Ishunina and Swaab, 2001). Changes in the activity of the nucleus basalis of Meynert modulate delta intensity in the occipital region via cholinergic pathways.

Low gamma oscillatory intensity in the occipital region also differed between these two phases. Interestingly, cross-frequency coupling studies suggest that gamma oscillations are modulated by low-frequency oscillations, such as delta and theta (Florin and Baillet, 2015). Thus, alterations in gamma activity may occur secondary to changes in the delta and theta bands. Overall, the source-level analysis revealed that the menstrual cycle most strongly influences theta oscillatory intensity, and that this change originates in the limbic system and deep structures such as the thalamus.

In addition, the source-level analysis revealed a trend in which the rostral (frontal/parietal) and caudal (temporal/occipital) regions exhibited reversal changes in oscillatory intensity between the proliferative and secretory phases (Figure 6). In most of the frequency bands, oscillatory intensities in rostral regions during the proliferative phase were higher than those during the secretory phase, while the inverse relationship was observed in caudal regions. Similar results were observed in our previous study regarding healthy ageing (Hoshi and Shigihara, 2020). In the previous study, we speculated that this inverse relationship may be explained by differences in the distribution of the dopaminergic system, which dominantly controls the rostral part of the brain (Alcaro et al., 2007). This may also explain the present result, as the dopaminergic system is affected by the menstrual cycle as well (Hidalgo-Lopez and Pletzer, 2017).

### Limitations

The study has five main limitations. First, we did not measure basal body temperature or levels of hormones such as oestrogen and progesterone due to ethical reasons. These factors are important and essential for identifying the phase of the menstrual cycle. However, these measurements place a substantial burden on participants, and many of them hesitated to mention the details of their menstrual cycle even in the clinic. Although the relationship between resting-state neural activity and the menstrual cycle has been investigated in previous studies, most of the results were based on subjective observations of EEG data. Therefore, we computed quantitative measures of resting-state neural activity and conducted the present study with a minimum setting to simulate the same conditions as clinical practice: recording neural oscillations non-invasively for 5 min and asking the participants to report the first day of the last MP. Despite this limited information, our results suggest that the influence of the menstrual cycle should be taken into account when functional neuroimaging is required in general practice settings. Second, the order of measurements between the MP and OP was not well balanced. MEG data from eight of 25 participants were acquired during the MP first, while data were acquired during the OP first in the remaining participants. To remove this potential confounding factor, the two conditions should have included an equal number of participants. In addition, the number of participants in the proliferative and secretory phases was not well matched due to the same ethical reasons. To overcome the potential bias, we applied a sophisticated statistical method, although we recognise that this is not the ideal approach. Nevertheless, our results were significant, supporting the notion that the menstrual cycle should be included as a control variable in future studies. Third, participants were recruited from a limited population: clinical staff in Kumagaya General Hospital. Thus, some variables (e.g., educational levels) may not have been the same as those in the general population. However, this conservative approach allowed us to easily confirm that all participants were healthy (Hoshi and Shigihara, 2020), which is not always possible when participants are sampled from the general population. Fourth, the study design was not identical between Comparison 1 (i.e., within-participant design) and Comparison 2 (i.e., between-participant design). The present research was primarily designed to investigate differences in neural oscillatory activity between the MP and OP (Comparison 1), as discrimination between the two phases is frequently necessary in clinical practice. We undertook an additional analysis (Comparison 2) to explore the mechanisms underlying these alterations, which thereby required a different study design. Fifth, to minimise the burden on research participants, we did not assess cognitive or emotional function using neuropsychological tests. Such tests may have been helpful in interpreting alterations in resting-state cortical activity. Although these analyses were beyond the scope of the present study, it may be interesting to include these variables along with basal body temperature and hormone levels in future studies.

## Conclusion

Resting-state neural oscillations are used as biomarkers for functional diseases, such as dementia (Poza et al., 2007; Hughes et al., 2019), epilepsy (Worrell et al., 2004; Lundstrom et al., 2019; Tanoue et al., 2021), and stroke (Sakamoto et al., 2016). However, accurate interpretation of clinical outcomes requires the identification and minimisation of potential confounding factors. Our findings suggest that spontaneous neural oscillations are affected by the menstrual cycle at both global and regional levels, as well as by age and sex. The changes elicited by the cycle are not limited to alpha rhythms, but also affect high- and low-frequency oscillations, particularly theta rhythms. The limbic system and parietal lobe play important roles in these changes in spontaneous neural oscillations. In conclusion, our findings suggest that the menstrual cycle should be considered to ensure accurate interpretation of functional neuroimaging in clinical practice, especially in the treatment of epilepsy and dementia. Future studies may wish to focus on the association between the frequency of epileptic seizures and the menstrual cycle (i.e., catamenial epilepsy) (Maguire and Nevitt, 2019) and on the interactions between the healthy menstrual cycle and dementia via hormonal systems (Moradi et al., 2018; Zhou et al., 2020).

## Data Availability Statement

The datasets presented in this study can be found in online repositories. The names of the repository/repositories and accession number(s) can be found below: Shigihara, Yoshihito, 2021, “Replication Data for: Healthy menstruation”, https://doi.org/10.7910/DVN/H4O0RE, Harvard Dataverse, V1.

## Ethics Statement

The studies involving human participants were reviewed and approved by the Ethics Committee of Kumagaya General Hospital. The patients/participants provided their written informed consent to participate in this study.

## Author Contributions

YS managed and designed the research project. RH and SI recruited participants. RH, SI, and KF scanned and managed MEG data. HH and YS analysed the data. HH, JP, VR-G, CG, and YS wrote the manuscript. KN and MH supervised the project as gynaecologists. All authors contributed to the article and approved the submitted version.

## Conflict of Interest

YS leads the joint research projects that were supported by RICOH Co., Ltd. HH was employed by RICOH Co., Ltd. VR-G received a PIF-UVa grant from the University of Valladolid. The remaining authors declare that the research was conducted in the absence of any commercial or financial relationships that could be construed as a potential conflict of interest.

## Publisher’s Note

All claims expressed in this article are solely those of the authors and do not necessarily represent those of their affiliated organizations, or those of the publisher, the editors and the reviewers. Any product that may be evaluated in this article, or claim that may be made by its manufacturer, is not guaranteed or endorsed by the publisher.

## Footnotes

1 https://www.fil.ion.ucl.ac.uk/spm/

2 https://www.hokuto7.or.jp/hospital/lang/english-home/meaw/

3 https://www.nitrc.org/projects/wfu_pickatlas/
