# Supplementary material for: The Menstrual Cycle Alters Resting-State Cortical Activity: A Magnetoencephalography Study
Source: Front Hum Neurosci. 2021 Jul 26;15:652789. doi: 10.3389/fnhum.2021.652789 (PMC8350571; doi:10.3389/fnhum.2021.652789)
Supplement: Supplementary file 1 [file Data_Sheet_1.pdf]

Supplementary Material

The Menstrual Cycle Alters Resting-State Cortical activity: A Magnetoencephalography Study

Rika Haraguchi[1†], Hideyuki Hoshi[2†], Sayuri Ichikawa[1†], Kohei Nakamura[3,4], Keisuke Fukasawa[1], Jesus Poza[5,6,7], Víctor Rodríguez-González[5,6], Carlos Gómez[5,6], Yoshihito Shigihara[2,8*]

[†]These authors have contributed equally to this work and shar first authorship.

[1]Clinical laboratory, Kumagaya General Hospital, Kumagaya City, Saitama, Japan

[2]Precision Medicine Centre, Hokuto Hospital, Obihiro City, Hokkaido, Japan

[3]Department of gynaecology, Kumagaya General Hospital, Kumagaya City, Saitama, Japan

[4]Genomics Unit, Keio Cancer Center, Keio University School of Medicine

[5]Biomedical Engineering Group, Higher Technical School of Telecommunications Engineering, University of Valladolid, Valladolid, Castilla y León, Spain

[6]Centro de Investigación Biomédica en Red en Bioingeniería, Biomateriales y Nanomedicina, (CIBER-BBN), Valladolid, Castilla y León, Spain

[7]Instituto de Investigación en Matemáticas (IMUVA), University of Valladolid, Valladolid, Castilla y León, Spain

[8]Precision Medicine Centre, Kumagaya General Hospital, Kumagaya City, Saitama, Japan

*** Correspondence:**
Yoshihito Shigihara
y-shigihara@hokuto7.or.jp

Supplementary Figure

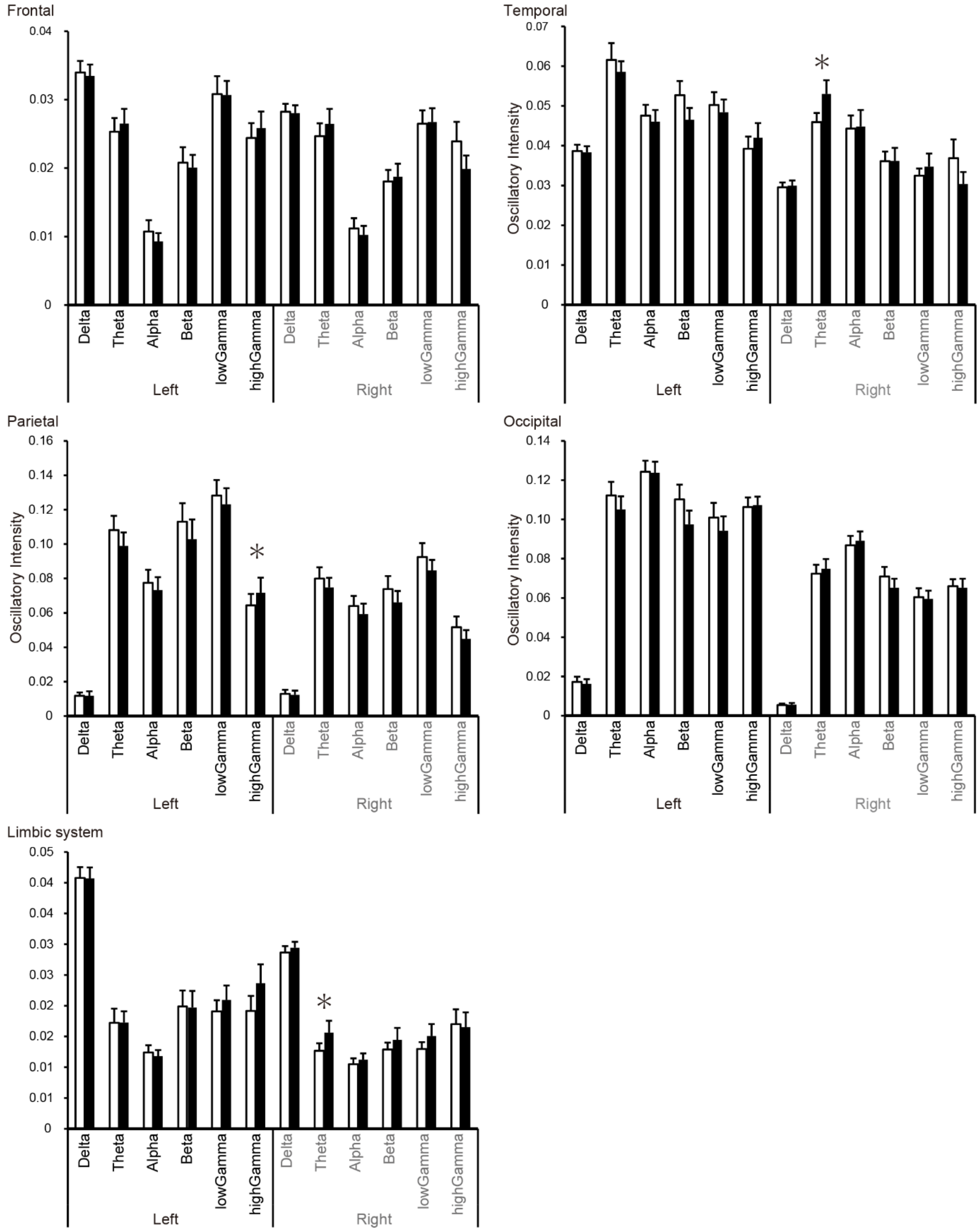

Supplementary Figure 1. Difference in oscillation intensity within ROI between menstrual period and out of the period. Open column, Menstrual period; Filled column, out of the period. Asterisks(*) indicate significant difference.
